# Somatic growth, aging, and longevity

**DOI:** 10.1038/s41514-017-0014-y

**Published:** 2017-09-29

**Authors:** Andrzej Bartke

**Affiliations:** Department of Internal Medicine, Southern Illinois University School of Medicine, 801N. Rutledge, P.O. Box 19628, Springfield, IL 62794-9628 USA

## Abstract

Although larger species of animals typically live longer than smaller species, the relationship of body size to longevity within a species is generally opposite. The longevity advantage of smaller individuals can be considerable and is best documented in laboratory mice and in domestic dogs. Importantly, it appears to apply broadly, including humans. It is not known whether theses associations represent causal links between various developmental and physiological mechanisms affecting growth and/or aging. However, variations in growth hormone (GH) signaling are likely involved because GH is a key stimulator of somatic growth, and apparently also exerts various “pro-aging” effects. Mechanisms linking GH, somatic growth, adult body size, aging, and lifespan likely involve target of rapamycin (TOR), particularly one of its signaling complexes, mTORC1, as well as various adjustments in mitochondrial function, energy metabolism, thermogenesis, inflammation, and insulin signaling. Somatic growth, aging, and longevity are also influenced by a variety of hormonal and nutritional signals, and much work will be needed to answer the question of why smaller individuals may be likely to live longer.

## Introduction

There is considerable evidence that developmental events, including pre-natal and post-natal growth can have a profound impact on adult phenotypes and risk of chronic disease. In this context, it is interesting to consider to what extent somatic growth and adult body size can influence the trajectory of aging and life expectancy. Pioneering studies of Samaras and his colleagues provided numerous examples of negative association of human height and lifespan.^1–3^ Other investigators emphasized a positive, rather than a negative, association of human stature with various health outcomes, including longevity.^4,5^ However, some of the reported associations are controversial.^6^ Importantly, studies from several laboratories reported reduced old age mortality and exceptional longevity in individuals with shorter stature and reduced somatotropic signaling.^7–10^ Evidence from genetic studies supports the negative association of somatotropic signaling and height with human longevity.^11,12^ A recent study revealed that the total 24-hour secretion of GH was lower in middle-aged offspring of long-lived families than in their partners.^13^

Deciphering relationships between growth and longevity in human populations is difficult because both can be powerfully influenced by environmental factors including nutrition, numerous public health measures, access to medical interventions and lifestyle factors such as smoking, alcohol, and drug use. In this article, we will briefly review evidence for the links between somatic growth, aging, and longevity in experimental animals and discuss the most likely underlying mechanisms.

## “Longevity genes” and somatic growth

The very exciting and largely unexpected discoveries of mutations that significantly extend longevity in a worm (*Caenorhabditis elegans*) or in a fly (*Drosophila melanogaster*)^14–17^ are an important part of the present understanding of the genetic control of aging. The striking effects of mutations of a single gene on longevity of heterothermic invertebrates led to the question of whether identifiable “longevity genes” also influence aging and lifespan in mammals, including our own species. Studies conducted during the last 25 years in laboratory stocks of house mice (*Mus musculus*) demonstrated that longevity of these animals can be extended by a natural loss-of-function mutation or targeted deletion of a single gene.^18–27^ With very few exceptions, the genetic modifications which produced a significant and reproducible extension of longevity in both females and males disrupted the so called “somatotropic axis,” that is, biosynthesis or action of the pituitary growth hormone (GH) and/or the insulin-like growth factor I (IGF-1), an important mediator of GH actions. Since in mammals the somatotropic axis is a key regulator of postnatal growth, these long-lived mutants exhibit major reductions in growth rate and in adult body size. It should be emphasized that the remarkably extended longevity of GH-deficient and GH-resistant mice is associated with multiple signs of delayed aging and with the extension of “healthspan,” the period of life free of functional deficits and chronic disease.^19–21,28^ This includes maintenance of normal cognitive function (learning and memory) into advanced age.^29,30^ The rate of aging of these long-lived mutants was initially reported to be either reduced or unaltered, with life extension being due to a delay, rather than a slowing, of age-related mortality.^31^ However, a more recent analysis using what we believe is a more pertinent methodology suggests that the rate of aging of long-lived, GH-deficient and GH-resistant mice is initially slower than in genetically normal (control) animals and accelerates only later in their life, after most of the control animals have died.^32^

Extended longevity and phenotypic characteristics of animals with deletion of genes acting downstream from the IGF-1 receptor such as IRS-1, IRS-2, or Akt^25–27^ and the well-documented anti-aging effects of calorie restriction, provide additional examples of the negative association of somatotropic signaling and growth with healthy aging and lifespan.

## Growth vs. longevity in transgenic and in genetically normal animals

The reciprocal relationship of longevity to growth rate and adult body size discovered in various dwarf mutants also applies to animals in which somatic growth and adult body size are experimentally enhanced. Transgenic mice, chronically expressing heterologous (in most cases bovine or human) GH in liver and other organs under control of metallothionein I or phosphoenolpyruvate carboxykinase promoters, grow faster than normal animals and achieve a greater adult body size, often exhibiting a very striking giant phenotype.^33,34^ These animals have much shorter lives than their normal siblings and exhibit multiple characteristics of early (premature) aging.^35^

Experimental animals with extreme phenotypes, such as encountered in genetic dwarfs and giant transgenics, are useful for identifying the underlying mechanisms and previously unsuspected physiological relationships. However, it is of obvious importance to determine whether the associations and causal relationships described in these animals apply to organisms that have not been genetically altered and to genetic and phenotypic variations within the normal range. In fact, the negative association of longevity with adult body weight has been demonstrated in comparisons of normal (“wild type”) animals form different stocks, inbred strains and selected lines in studies going back to the ‘60 s^35–37^ and, more recently, in comparisons of individual animals from a normal, genetically heterogeneous population.^38^

Importantly, the negative association of body size and longevity extends to other mammalian species, with differences in longevity between different breeds of domestic dogs, and between individual dogs differing in size providing the most striking example.^39,40^ It is interesting, but currently difficult to explain, why these relationships within species are opposite to the fairly consistent trend for large species of mammals and birds living longer than smaller species. One could speculate that because smaller species are more vulnerable to predation and other environmental hazards, they have developed life-course strategies for early reproduction and high fecundity. This, in turn, may divert available resources away from repair and maintenance and thus lead to a shorter lifespan. The surprising tendency of circulating IGF-1 levels to be lower, rather than higher, in large species^41^ may also prove to be a contributing factor to their longevity.

## Search for mechanisms linking somatic growth with aging and longevity

**Fig. 1:**
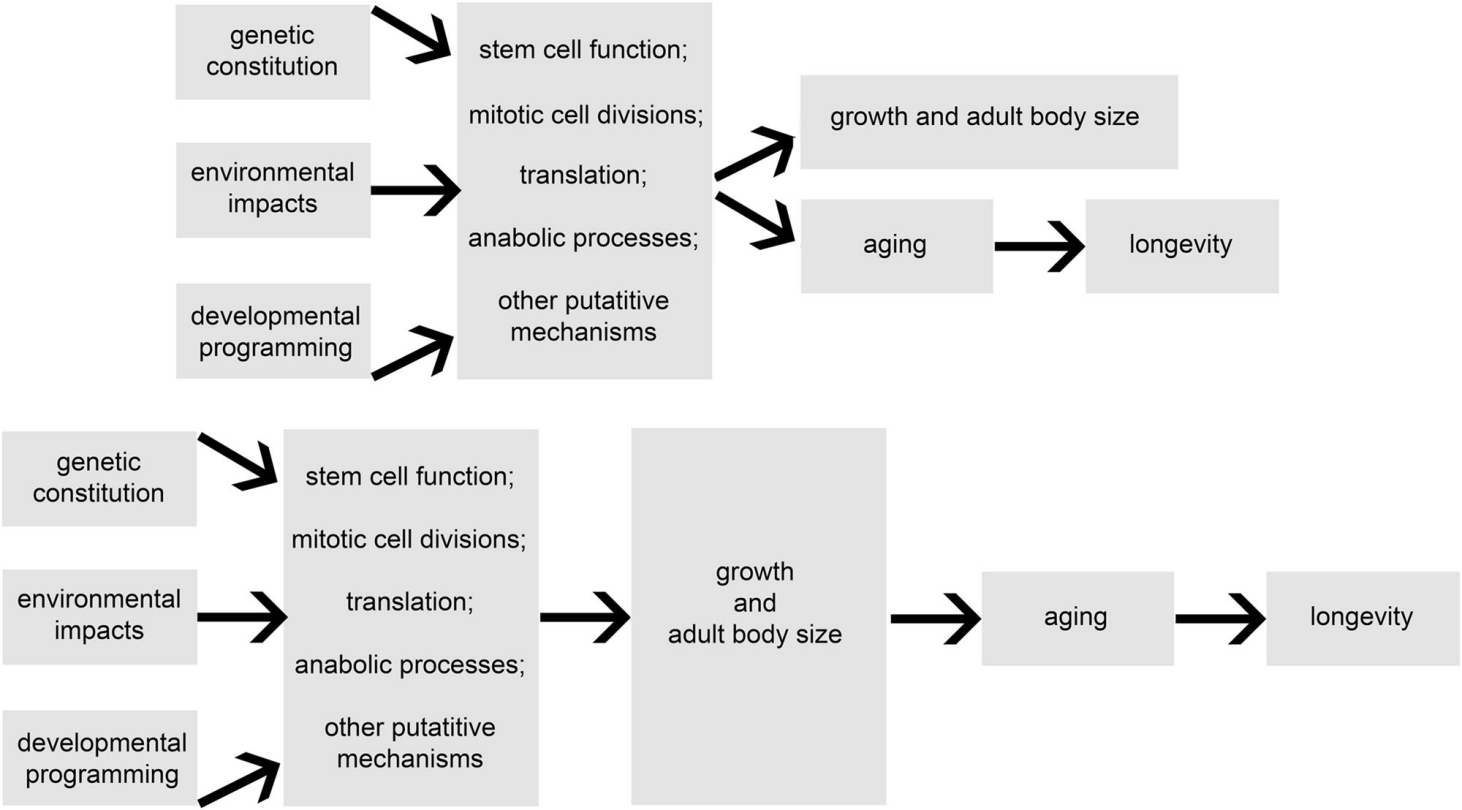
Negative association of adult body size and longevity may result from impact of the underlying metabolic process on both somatic growth and aging (top panel) or, presumably less likely, from the negative effects of growth and adult body size on the rate of aging and longevity (bottom panel)

The (very consistent) negative association of adult body size and longevity in laboratory mice and other mammalian species brings up the questions of causality and underlying mechanisms. Available data can be interpreted as evidence that normal growth involves some intrinsic “costs” in terms of aging and longevity. Thus, faster or longer growth, and the consequent attainment of greater body size, somehow predispose the organism to earlier and/or faster aging and a shorter lifespan. The underlying mechanisms could be envisaged to influence growth with a secondary impact on aging and lifespan, or to independently influence both growth and aging, possibly via different signaling pathways or cellular processes (Fig. 1). In either case, identifying the mechanisms involved is an important goal of our research and work in other laboratories. Comparing gene expression and phenotypic characteristics of long-lived mutants which have various defects in the somatotropic axis with the same characteristics of age-matched and sex-matched normal animals from the same strain identified a number of suspected mechanisms of extended longevity.^19,21^ Defining the role of these mechanisms in the extension of healthspan and lifespan in the examined mutants is complicated by the fact that many of the differences between mutant and control (“wild type”) animals could represent either mechanisms or markers of delayed and/or slower aging. For example, long-lived Ames dwarf and growth hormone receptor knockout (GHRKO) mice are more insulin sensitive than their normal siblings^42^ but insulin sensitivity generally declines during aging.^43,44^ Thus, this difference could simply confirm the fact that at the same chronological age, the long-lived dwarf mice are biologically younger. This difficult issue was addressed in one of our studies by comparing gene expression data in long-lived mutants to two groups of normal animals that were either of the same chronological age or a comparable “biological age,” that is their age represented a similar percent of their life expectancy.^45^ In this particular study, differences in hepatic expression of the examined genes in GHRKO vs. normal mice were shown to be due to the differences in genotype rather than in biological age.^45^ However, we are not aware of a similar analysis being performed in other long-lived mutants or in studies examining other candidate mechanisms of aging.

Detailed discussion of all the mechanisms that appear to be involved in linking reduced somatotropic signaling to slower aging and extended longevity is outside the scope of this review. Interested readers are referred to several recent review articles.^19–21^ Mechanisms most likely related to the negative association of somatic growth and longevity and to the opposite effects of GH signaling on these processes are briefly discussed below.

## Mechanistic target of rapamycin (mTOR)

The ability of GH to directly or indirectly activate the mTOR signaling pathway provides one of the most likely explanations of how this hormone can exert positive effects on somatic growth and negative effects on the lifespan. Mechanistic TOR (mTOR) pathway integrates somatotropic, nutritional and stress signals and plays a role in the control of autophagy and cell senescence.^46^ While activation of mTOR signaling prevents cell death, promotes protein synthesis, growth and cell divisions, it apparently also accelerates aging. Blagosklonny and his colleagues suggested that aging can be viewed as “a continuation of developmental growth driven by genetic pathways such as mTOR”.^47^ These investigators also proposed that gender differences in longevity of mice may be related to greater mTOR activity in males^48^ and that conversion of reversible cell cycle arrest to senescence (termed “geroconversion”) represents one of the mechanisms by which mTOR promotes aging.^49^ The excess “developmental” growth driven by mTOR presumably contributes to the accumulation of unfolded proteins, endoplasmic reticulum (ER) stress, and reactive oxygen species (ROS) production, and leads to inhibition of autophagy and promotion of cell senescence.

Pharmacological or genetic suppression of mTOR signaling pathways results in extended longevity in organisms ranging from yeast to mice^50–52^ mTOR exerts its function via two complexes: mTORC1 and mTORC2. There is increasing evidence that the “anti-aging” effect of mTOR suppression is due to inhibition of mTORC1 signaling. For example, long-lived mice with disrupted somatotropic axis have diminished TORC1 signaling, while TORC2 activity may be increased^53–55^ mTORC2 appears to act to prevent, rather than promote aging, and consequently, its inhibition can reduce longevity. The evidence for anti-aging effects of mTORC2 signaling includes demonstration that genetic deletion of Rictor, a mediator of TORC2 signaling, reduces longevity in male mice.^56^ Interestingly, body weight and accumulation of white adipose tissue (WAT) in these animals were increased, suggesting that mTORC2, in contrast to mTORC1, may normally act as a negative regulator of growth.^57^ Further support of the role of TORC-2 in the control of aging, adipose-specific deletion of Rictor leads to increases in body size, weight of non-adipose organs and levels of IGF-1, IGFBP3, and insulin with a concomitant decrease of adiponectin, i.e., produces phenotypic characteristics generally associated with accelerated aging.^57^

## Energy metabolism and thermogenesis

Another mechanism which may be contributing to the longevity benefits of small body size involves adjustments in mitochondrial function and energy metabolism in response to greater heat loss and increased demand for thermogenesis. Smaller individuals have a greater body surface to body mass ratio and, thus, lose heat faster. Therefore, they have to increase thermogenesis to maintain body temperature. This physiological response is of particular significance in laboratory mice whose thermoneutral temperature is ~30 °C (86 °F) and, thus, housing at a “standard” animal room temperature (~22 °C, or 72 °F) represents a chronic mild cold stress.^58^ ^–60^ The long-lived diminutive GHRKO and hypopituitary dwarf mice, in fact, exhibit increases in the mass and activity of brown adipose tissue (BAT), the key site of non-shivering thermogenesis, along with evidence for thermogenic activation, the so-called “browning” of WAT (Ref. 61, Darcy and Bartke, in press). Increased energy demand for maintaining a normal [or slight reduced,^62^ body temperature in these animals is reflected in increased consumption of oxygen per gram of body weight^63^ and is accompanied by a marked reduction in respiratory quotient (RQ, equivalent to respiratory exchange ratio).^63^ Reduced RQ implies increased reliance on fatty acids as a metabolic fuel which is generally considered a marker of improved mitochondrial efficiency leading to generation of smaller amounts of noxious ROS.^64–67^ In fact, reduced RQ in long-lived dwarf mice is associated with increased expression of genes involved in β-oxidation of free fatty acids (Sun L, Darcy J, and Bartke A, unpublished) and with reduced ROS generation.^68^ These alterations, together with improved anti-oxidant defenses^18,69^ probably account for less oxidative damage to DNA and other macromolecules in these long-lived animals.^69–71^ Although it remains to be conclusively proven which of these associations are causally related to aging, we believe that improvements in mitochondrial function in response to increased demand for thermogenesis represent one of the mechanisms linking reductions in somatotropic signaling, growth and adult body size to slower (and/or delayed) aging and extended longevity.^72,73^

## Indirect links between somatic growth and longevity

In addition to the mechanisms discussed above, the negative association of body size and longevity within a species likely reflects several indirect, but important, mechanisms. These include pleiotropic actions of GH which affect both body size and aging, acting through different, but likely physiologically related mechanisms. Growth hormone is a key regulator of hepatic IGF-1 expression, circulating IGF-1 levels, growth and adult body size, but it also appears to promote aging by a variety of actions such as inducing insulin resistance^35,42^ promoting cell senescence^74^ and chronic low grade inflammation,^75,76^ as well as favoring differentiation and depletion of at least one type of stem cell.^77^ Thus, the spectrum of known biological actions of GH could explain why growth and adult body size would tend to be negatively associated with longevity.^21,78^

Another indirect link between body size and longevity concerns alterations in disease risk. Growth hormone and IGF-1, as well as stature, have been related to cancer incidence on the basis of numerous in vitro, in vivo and epidemiological studies.^79–81^ Severe GH resistance in the syndrome of Laron dwarfism produces a remarkable degree of protection from cancer.^82^ Neoplastic disease may have little if any influence on aging, but it has an obvious and major impact on longevity. This is particularly striking in laboratory mice in which cancer is an important and, in many strains, by far the most common cause of death. The fact that GH induces insulin resistance, an important element of the metabolic syndrome, prediabetes and diabetes, should also be mentioned in this context. The risk of diabetes is increased in association with abnormally elevated GH levels in acromegaly^83^ and reduced in the GH-resistant individuals with Laron syndrome.^84^

Impacts of nutrition on growth, body composition, disease risk and aging are outside of the scope of this brief article, but can provide yet another indirect mechanism for the observed associations. Thus, both pre-natal and post-natal overnutrition can stimulate growth, as well as obesity and also increase risk of various chronic diseases in adulthood.^85,86^ Rapid growth in response to food availability after a period of nutrient shortage, the so-called “catch-up growth”, may be particularly important in this context.^87–89^ Increased risk of chronic disease in adult life can obviously reduce longevity and it also may represent a symptom of accelerated aging.^90,91^ Results of recent and ongoing studies in our laboratory indicate that treatment of hypopituitary Ames dwarf mice with GH injections during a relatively brief period (6 weeks) during development increases adult body size and significantly reduces the remarkably long lifespan of these animals.^92,93^ Importance of early (peripubertal) GH actions in the control of longevity is indirectly supported by the recent report that disruption of the GH receptor gene in adult mice produces a relatively modest increase in lifespan only in females.^94^

It should also be mentioned that the endocrine basis of the complex relationships between growth-related processes and aging is not limited to the somatotropic axis. Ames and Snell dwarfs, which represent some of the extremes of mouse longevity, are both GH- and thyroid stimulating hormone-deficient and, thus, profoundly hypothyroid.^18,19^ Chronic thyroxine treatment can shorten longevity of Snell dwarf mice,^95^ as well as normal rats ^96^ while subclinical hypothyroidism was associated with exceptional longevity in women.^97,98^ However, treatment of dwarf mice with thyroxine limited to the peripubertal period did not alter their longevity.^93,99^

The relationships between the activity of the hypothalamic-pituitary-adrenal axis and aging are complex and poorly understood.^100^ However, chronic elevation of glucocorticoid levels can promote age-related pathology, and is suspected of contributing to accelerated aging of GH transgenic mice.^101^

Much more work will be needed to determine whether the association of reduced body size with extended longevity is truly causal, and to identify the mechanisms underlying this association.

## Conclusions

Negative association of longevity with GH signaling, somatic growth and adult body size discovered in GH-deficient and GH-resistant mutant mice applies also to genetically normal individuals and to other mammalian species. Stimulation of mTORC1 singling by the somatotropic axis could explain this association but many other mechanisms appear to be involved.

## References

[1] Samaras TT, Storms LH (1992). Impact of height and weight on life span. Bull. World Health Organ..

[2] Samaras TT, Elrick H (1999). Height, body size and longevity. Acta Med. Okayama.

[3] Samaras, T. T. *Human body size and the laws of scaling: physiological, performance, growth, longevity and ecological ramifications*. (Nova Science Publishers, Inc., 1st edn, 2007), pp. 381.

[4] Paajanen TA, Oksala NK, Kuukasjarvi P, Karhunen PJ (2010). Short stature is associated with coronary heart disease: a systematic review of the literature and a meta-analysis. Eur. Heart J..

[5] Nelson CP (2015). Genetically determined height and coronary artery disease. N. Engl. J. Med..

[6] Nuesch E (2015). Adult height, coronary heart disease and stroke: a multi-locus Mendelian randomization meta-analysis. Int. J. Epidemiol..

[7] van Heemst D (2005). Reduced insulin/IGF-1 signalling and human longevity. Aging Cell.

[8] Suh Y (2008). Functionally significant insulin-like growth factor I receptor mutations in centenarians. Proc. Natl Acad. Sci. USA.

[9] He Q (2014). Shorter men live longer: association of height with longevity and FOXO3 genotype in American men of Japanese ancestry. PLoS One.

[10] Milman S (2014). Low insulin-like growth factor-1 level predicts survival in humans with exceptional longevity. Aging Cell.

[11] Teumer A (2016). Genomewide meta-analysis identifies loci associated with IGF-I and IGFBP-3 levels with impact on age-related traits. Aging Cell.

[12] De Luca M, Crocco P, De Rango F, Passarino G, Rose G (2016). Association of the Laminin, Alpha 5 (LAMA5) rs4925386 with height and longevity in an elderly population from Southern Italy. Mech. Ageing Dev..

[13] van der Spoel E (2016). Growth hormone secretion is diminished and tightly controlled in humans enriched for familial longevity. Aging Cell.

[14] Johnson TE, Conley WL, Keller ML (1988). Long-lived lines of *Caenorhabditis elegans* can be used to establish predictive biomarkers of aging. Exp. Gerontol..

[15] Kenyon C, Chang J, Gensch E, Rudner A, Tabtiang R (1993). A *C. elegans* mutant that lives twice as long as wild type. Nature.

[16] Tatar M, Bartke A, Antebi A (2003). The endocrine regulation of aging by insulin-like signals. Science.

[17] Clancy DJ (2001). Extension of life-span by loss of CHICO, a *Drosophila* insulin receptor substrate protein. Science.

[18] Brown-Borg HM (2015). The somatotropic axis and longevity in mice. Am. J. Physiol. Endocrinol. Metab..

[19] Bartke A (2011). Single-gene mutations and healthy ageing in mammals. Philos. Trans. R. Soc. Lond. B. Biol. Sci..

[20] Junnila RK, List EO, Berryman DE, Murrey JW, Kopchick JJ (2013). The GH/IGF-1 axis in ageing and longevity. Nat. Rev. Endocrinol..

[21] Bartke A, Sun LY, Longo V (2013). Somatotropic signaling: trade-offs between growth, reproductive development, and longevity. Physiol. Rev..

[22] Flurkey K, Papaconstantinou J, Miller RA, Harrison DE (2001). Lifespan extension and delayed immune and collagen aging in mutant mice with defects in growth hormone production. Proc. Natl Acad. Sci. USA.

[23] Coschigano KT (2003). Deletion, but not antagonism, of the mouse growth hormone receptor results in severely decreased body weights, insulin, and insulin-like growth factor I levels and increased life span. Endocrinology.

[24] Conover CA (2010). PAPP-A: a new anti-aging target?. Aging Cell.

[25] Selman C, Partridge L, Withers DJ (2011). Replication of extended lifespan phenotype in mice with deletion of insulin receptor substrate 1. PLoS One.

[26] Taguchi A, White MF (2008). Insulin-like signaling, nutrient homeostasis, and life span. Annu. Rev. Physiol..

[27] Nojima A (2013). Haploinsufficiency of akt1 prolongs the lifespan of mice. PLoS One.

[28] Ikeno Y (2009). Reduced incidence and delayed occurrence of fatal neoplastic diseases in growth hormone receptor/binding protein knockout mice. J. Gerontol. A. Biol. Sci. Med. Sci..

[29] Kinney BA, Meliska CJ, Steger RW, Bartke A (2001). Evidence that Ames dwarf mice age differently from their normal siblings in behavioral and learning and memory parameters. Horm. Behav..

[30] Kinney BA, Coschigano KT, Kopchick JJ, Steger RW, Bartke A (2001). Evidence that age-induced decline in memory retention is delayed in growth hormone resistant GH-R-KO (Laron) mice. Physiol. Behav..

[31] de Magalhaes JP, Cabral JA, Magalhaes D (2005). The influence of genes on the aging process of mice: a statistical assessment of the genetics of aging. Genetics.

[32] Koopman JJ (2016). Measuring aging rates of mice subjected to caloric restriction and genetic disruption of growth hormone signaling. Aging.

[33] Palmiter RD (1982). Dramatic growth of mice that develop from eggs microinjected with metallothionein-growth hormone fusion genes. Nature.

[34] McGrane MM (1990). Metabolic effects of developmental, tissue-, and cell-specific expression of a chimeric phosphoenolpyruvate carboxykinase (GTP)/bovine growth hormone gene in transgenic mice. J. Biol. Chem..

[35] Bartke A (2003). Can growth hormone (GH) accelerate aging? Evidence from GH-transgenic mice. Neuroendocrinology.

[36] Roberts RC (1961). The lifetime growth and reproduction of selected strains of mice. Heredity.

[37] Eklund J, Bradford GE (1977). Longeveity and lifetime body weight in mice selected for rapid growth. Nature.

[38] Miller RA, Harper JM, Galecki A, Burke DT (2002). Big mice die young: early life body weight predicts longevity in genetically heterogeneous mice. Aging Cell.

[39] Patronek GJ, Waters DJ, Glickman LT (1997). Comparative longevity of pet dogs and humans: implications for gerontology research. J. Gerontol. A. Biol. Sci. Med. Sci..

[40] Greer KA, Canterberry SC, Murphy KE (2007). Statistical analysis regarding the effects of height and weight on life span of the domestic dog. Res. Vet. Sci..

[41] Stuart JA, Page MM (2010). Plasma IGF-1 is negatively correlated with body mass in a comparison of 36 mammalian species. Mech. Ageing Dev..

[42] Masternak MM, Panici JA, Bonkowski MS, Hughes LF, Bartke A (2009). Insulin sensitivity as a key mediator of growth hormone actions on longevity. J. Gerontol. A. Biol. Sci. Med. Sci..

[43] DeFronzo RA (1981). Glucose intolerance and aging. Diabetes Care.

[44] Xu C (2013). Selective overexpression of human SIRT1 in adipose tissue enhances energy homeostasis and prevents the deterioration of insulin sensitivity with ageing in mice. Am. J. Transl. Res..

[45] Panici JA (2009). Is altered expression of hepatic insulin-related genes in growth hormone receptor knockout mice due to GH resistance or a difference in biological life spans?. J. Gerontol. A. Biol. Sci. Med. Sci..

[46] Lees H, Walters H, Cox LS (2016). Animal and human models to understand ageing. Maturitas.

[47] Blagosklonny MV (2013). Aging is not programmed: genetic pseudo-program is a shadow of developmental growth. Cell Cycle.

[48] Leontieva OV, Paszkiewicz GM, Blagosklonny MV (2012). Mechanistic or mammalian target of rapamycin (mTOR) may determine robustness in young male mice at the cost of accelerated aging. Aging.

[49] Blagosklonny MV (2014). Geroconversion: irreversible step to cellular senescence. Cell Cycle.

[50] Harrison DE (2009). Rapamycin fed late in life extends lifespan in genetically heterogeneous mice. Nature.

[51] Johnson SC, Rabinovitch PS, Kaeberlein M (2013). mTOR is a key modulator of ageing and age-related disease. Nature.

[52] McCormick MA, Tsai SY, Kennedy BK (2011). TOR and ageing: a complex pathway for a complex process. Philos. Trans. R. Soc. Lond. B. Biol. Sci..

[53] Sharp ZD, Bartke A (2005). Evidence for down-regulation of phosphoinositide 3-kinase/Akt/mammalian target of rapamycin (PI3K/Akt/mTOR)-dependent translation regulatory signaling pathways in Ames dwarf mice. J. Gerontol. A. Biol. Sci. Med. Sci..

[54] Dominick G (2015). Regulation of mTOR activity in Snell dwarf and GH receptor gene-disrupted mice. Endocrinology.

[55] Lamming DW (2012). Rapamycin-induced insulin resistance is mediated by mTORC2 loss and uncoupled from longevity. Science.

[56] Lamming DW (2014). Depletion of Rictor, an essential protein component of mTORC2, decreases male lifespan. Aging Cell.

[57] Cybulski N, Polak P, Auwerx J, Ruegg MA, Hall MN (2009). mTOR complex 2 in adipose tissue negatively controls whole-body growth. Proc. Natl Acad. Sci. USA.

[58] Gordon CJ (2012). Thermal physiology of laboratory mice: definining thermoneutrality. J. Therm. Biol..

[59] Karp CL (2012). Unstressing intemperate models: how cold stress undermines mouse modeling. J. Exp. Med..

[60] Maloney SK, Fuller A, Mitchell D, Gordon C, Overton JM (2014). Translating animal model research: does it matter that our rodents are cold?. Physiol..

[61] Li Y, Knapp JR, Kopchick JJ (2003). Enlargement of interscapular brown adipose tissue in growth hormone antagonist transgenic and in growth hormone receptor gene-disrupted dwarf mice. Exp. Biol. Med..

[62] Hunter WS, Croson WB, Bartke A, Gentry MV, Meliska CJ (1999). Low body temperature in long-lived Ames dwarf mice at rest and during stress. Physiol. Behav..

[63] Westbrook R, Bonkowski MS, Arum O, Strader AD, Bartke A (2014). Metabolic alterations due to caloric restriction and every other day feeding in normal and growth hormone receptor knockout mice. J. Gerontol. A. Biol. Sci. Med. Sci..

[64] Anderson RM, Weindruch R (2010). Metabolic reprogramming, caloric restriction and aging. Trends Endocrinol. Metab..

[65] Perdomo G (2004). Increased beta-oxidation in muscle cells enhances insulin-stimulated glucose metabolism and protects against fatty acid-induced insulin resistance despite intramyocellular lipid accumulation. J. Biol. Chem..

[66] Gonzalez-Covarrubias V (2013). Lipidomics of familial longevity. Aging Cell.

[67] Orellana-Gavalda JM (2011). Molecular therapy for obesity and diabetes based on a long-term increase in hepatic fatty-acid oxidation. Hepatology.

[68] Brown-Borg HM, Johnson WT, Rakoczy SG (2012). Expression of oxidative phosphorylation components in mitochondria of long-living Ames dwarf mice. Age.

[69] Brown-Borg HM, Bode AM, Bartke A (1999). Antioxidative mechanisms and plasma growth hormone levels: potential relationship in the aging process. Endocrine.

[70] Brown-Borg HM, Rakoczy SG (2000). Catalase expression in delayed and premature aging mouse models. Exp. Gerontol..

[71] Sanz A, Bartke A, Barja G (2002). Long-lived Ames dwarf mice: oxidative damage to mitochondrial DNA in heart and brain. J. Am. Aging Assoc..

[72] Brown-Borg HM, Bartke A (2012). GH and IGF1: roles in energy metabolism of long-living GH mutant mice. J. Gerontol. A. Biol. Sci. Med. Sci..

[73] Westbrook R. Ph.D. *The effects of altered growth hormone signaling on murine metabolism*. Dissertation, Southern Illinois University (2012).

[74] Stout MB (2014). Growth hormone action predicts age-related white adipose tissue dysfunction and senescent cell burden in mice. Aging.

[75] Bartke A (2016). Healthspan and longevity can be extended by suppression of growth hormone signaling. Mamm. Genome.

[76] Sadagurski M (2015). Growth hormone modulates hypothalamic inflammation in long-lived pituitary dwarf mice. Aging Cell.

[77] Ratajczak J (2011). Higher number of stem cells in the bone marrow of circulating low Igf-1 level Laron dwarf mice--novel view on Igf-1, stem cells and aging. Leukemia.

[78] Bartke A, List EO, Kopchick JJ (2016). The somatotropic axis and aging: Benefits of endocrine defects. Growth Horm. Igf. Res..

[79] Green J (2011). Height and cancer incidence in the Million Women Study: prospective cohort, and meta-analysis of prospective studies of height and total cancer risk. Lancet Oncol..

[80] Batty GD (2010). Adult height and cancer mortality in Asia: the Asia Pacific Cohort Studies Collaboration. Ann. Oncol..

[81] C. Emerging Risk Factors. (2012). Adult height and the risk of cause-specific death and vascular morbidity in 1 million people: individual participant meta-analysis. Int. J. Epidemiol..

[82] Laron Z (2008). The GH-IGF1 axis and longevity. The paradigm of IGF1 deficiency. Hormones-Athens.

[83] Mercado M (2014). Successful mortality reduction and control of comorbidities in patients with acromegaly followed at a highly specialized multidisciplinary clinic. J. Clin. Endocrinol. Metab..

[84] Guevara-Aguirre J (2011). Growth hormone receptor deficiency is associated with a major reduction in pro-aging signaling, cancer, and diabetes in humans. Sci. Transl. Med.

[85] Ozanne SE, Fernandez-Twinn D, Hales CN (2004). Fetal growth and adult diseases. Semin. Perinatol..

[86] Blackmore HL, Ozanne SE (2013). Maternal diet-induced obesity and offspring cardiovascular health. J. Dev. Orig. Health Dis..

[87] Hales CN, Ozanne SE (2003). The dangerous road of catch-up growth. J. Physiol..

[88] Eriksson JG (1999). Catch-up growth in childhood and death from coronary heart disease: longitudinal study. BMJ.

[89] Cettour-Rose P (2005). Redistribution of glucose from skeletal muscle to adipose tissue during catch-up fat: a link between catch-up growth and later metabolic syndrome. Diabetes.

[90] Kirkland JL (2002). The biology of senescence: potential for prevention of disease. Clin. Geriatr. Med..

[91] Kennedy BK (2014). Geroscience: linking aging to chronic disease. Cell.

[92] Bartke A (2015). Early life events can shape aging and longevity. Curr. Aging Sci..

[93] Panici JA (2010). Early life growth hormone treatment shortens longevity and decreases cellular stress resistance in long-lived mutant mice. FASEB J..

[94] Junnila RK (2016). Disruption of the growth hormone receptor gene in adult mice increases maximal lifespan in females. Endocrinology.

[95] Vergara M, Smith-Wheelock M, Harper JM, Sigler R, Miller RA (2004). Hormone-treated snell dwarf mice regain fertility but remain long lived and disease resistant. J. Gerontol. A. Biol. Sci. Med. Sci..

[96] Ooka H, Shinkai T (1986). Effects of chronic hyperthyroidism on the lifespan of the rat. Mech. Ageing Dev..

[97] Rozing MP (2010). Familial longevity is associated with decreased thyroid function. J. Clin. Endocrinol. Metab..

[98] Bowers J (2013). Thyroid hormone signaling and homeostasis during aging. Endocr. Rev..

[99] Darcy J (2016). Original Research: metabolic alterations from early life thyroxine replacement therapy in male Ames dwarf mice are transient. Exp. Biol. Med..

[100] Gupta D, Morley JE (2014). Hypothalamic-pituitary-adrenal (HPA) axis and aging. Compr. Physiol..

[101] Cecim M, Alvarez-Sanz M, Van de Kar L, Milton S, Bartke A (1996). Increased plasma corticosterone levels in bovine growth hormone (bGH) transgenic mice: effects of ACTH, GH and IGF-I on in vitro adrenal corticosterone production. Transgenic Res..

